# Supplementary figures and images for: Markov Chain Abstractions of Electrochemical Reaction-Diffusion in Synaptic Transmission for Neuromorphic Computing
Source: Front Neurosci. 2021 Nov 29;15:698635. doi: 10.3389/fnins.2021.698635 (PMC8667025; doi:10.3389/fnins.2021.698635)

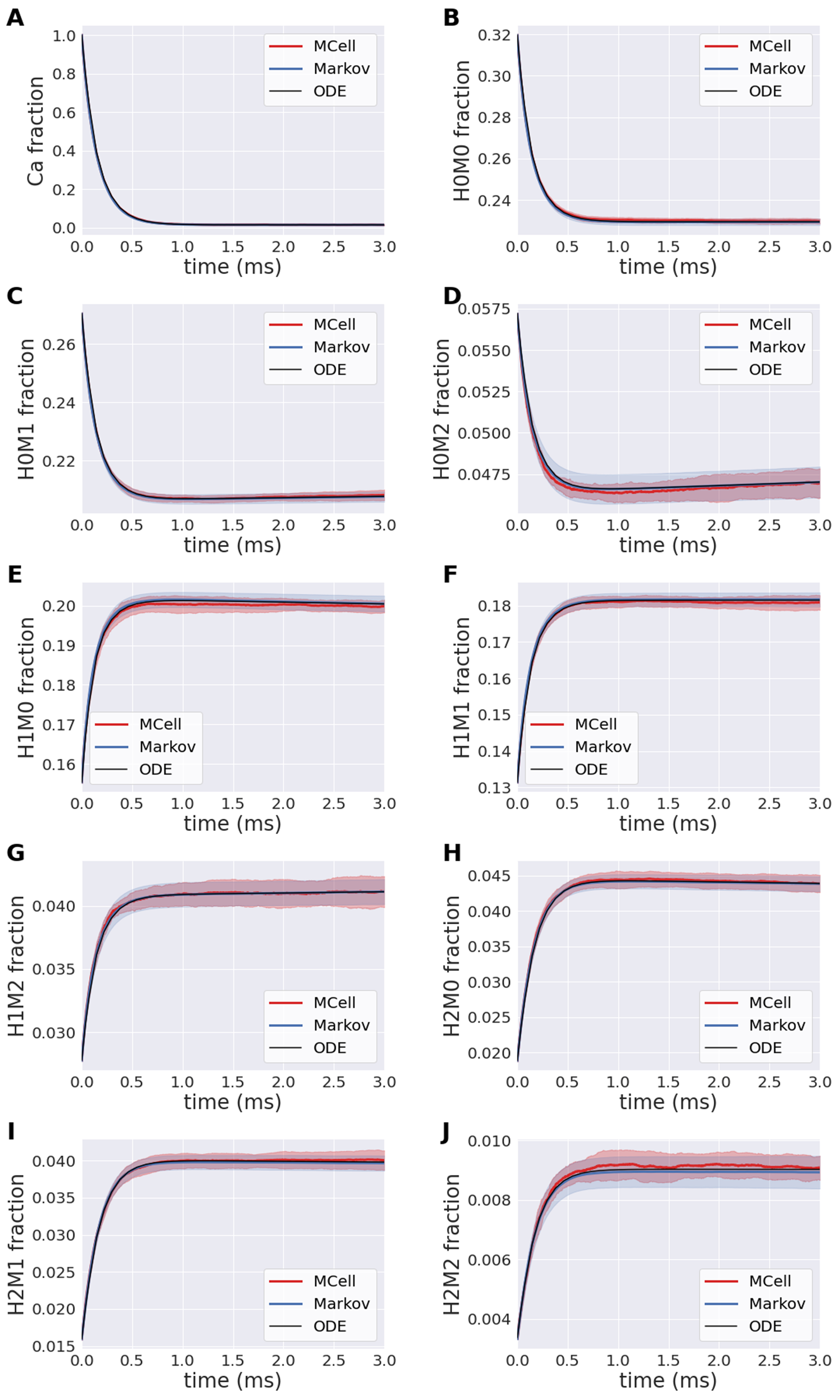

Supplement: Supplementary file 1 [file Image_1.JPEG]
